# Effects of *Spartina alterniflora* Invasion on Soil Respiration in the Yangtze River Estuary, China

**DOI:** 10.1371/journal.pone.0121571

**Published:** 2015-03-23

**Authors:** Naishun Bu, Junfeng Qu, Zhaolei Li, Gang Li, Hua Zhao, Bin Zhao, Bo Li, Jiakuan Chen, Changming Fang

**Affiliations:** 1 Coastal Ecosystems Research Station of the Yangtze River Estuary, Ministry of Education Key Laboratory for Biodiversity Science and Ecological Engineering, The Institute of Biodiversity Science, Fudan University, Shanghai, China; 2 School of Environmental Science and Spatial Informatics, China University of Mining and Technology, Xuzhou, China; 3 Low-carbon Energy Research Institute, China University of Mining and Technology, Xuzhou, China; The University of Auckland, NEW ZEALAND

## Abstract

Many studies have found that plant invasion can enhance soil organic carbon (SOC) pools, by increasing net primary production (NPP) and/or decreased soil respiration. While most studies have focused on C input, little attention has been paid to plant invasion effects on soil respiration, especially in wetland ecosystems. Our study examined the effects of *Spartina alterniflora* invasion on soil respiration and C dynamics in the Yangtze River estuary. The estuary was originally occupied by two native plant species: *Phragmites australis* in the high tide zone and *Scirpus mariqueter* in the low tide zone. Mean soil respiration rates were 185.8 and 142.3 mg CO_2_ m^−2^ h^−1^ in *S*. *alterniflora* and *P*. *australis* stands in the high tide zone, and 159.7 and 112.0 mg CO_2_ m^−2^ h^−1^ in *S*. *alterniflora* and *S*. *mariqueter* stands in the low tide zone, respectively. Aboveground NPP (ANPP), SOC, and microbial biomass were also significantly higher in the *S*. *alterniflora* stands than in the two native plant stands. *S*. *alterniflora* invasion did not significantly change soil inorganic carbon or pH. Our results indicated that enhanced ANPP by *S*. *alterniflora* exceeded invasion-induced C loss through soil respiration. This suggests that *S*. *alterniflora* invasion into the Yangtze River estuary could strengthen the net C sink of wetlands in the context of global climate change.

## Introduction

Biological invasion is one of the most pervasive elements of global change [1]. Many exotic species are invading new regions at an unprecedented rate due to human activities [2]. Plant invasion threatens the biodiversity and stability of native ecosystems, alters ecosystem functions and processes, and changes ecosystem carbon (C) cycles [3, 4]. Many studies have found that plant invasion significantly enhances soil organic carbon (SOC) pools and net primary production (NPP) [5]. Increased SOC pools in the invaded ecosystems could result from increased NPP and/or decreased soil respiration. Most studies have investigated C input into the soils affected by plant invasion (e.g., NPP) [2, 5]. Little attention has been paid to the impact of plant invasion on soil respiration; this factor is important because soil respiration is the largest terrestrial source of atmospheric CO_2_ (68–80 Pg C yr^-1^) in the global C cycle [6]. The current lack of research limits our understanding of invasion-effects on ecosystem C cycles.

Recent studies suggest that plant invasion could substantially impact soil respiration; however, results have been somewhat controversial, with positive, negative, or negligible effects [7, 8]. These discrepancies have largely resulted from differences in the factors controlling soil respiration in different field conditions [9, 10]. For example, soil respiration significantly increased after woody plant invasion into southern Texas and northern Mexico grasslands, likely due to increased SOC [7]. Conversely, soil respiration decreased in eastern Kansas, largely because of lower soil temperature under woody plants [8]. A separate global synthesis, with only a few in situ field measurements, suggested that soil respiration was not significantly affected by woody plant invasion [11]. These studies mainly focused on the effects of woody plant invasion into grasslands. Little work on soil respiration has been done in other ecosystems, especially in wetland ecosystems. Wetlands are important C pools in terrestrial ecosystems, and are particularly vulnerable to global change [12].

Wetlands occupy 4–6% of the earth’s land, but contain approximately 33% of global terrestrial soil C [13]. Wetlands are sinks of exogenous nutrients and propagules, increasing their vulnerability to plant invasion [14]. Previous studies have reported that plant invasion impacts many features of wetlands, including plant species composition, microbial activities, and soil properties [3, 15]. Invasive plants may regulate soil respiration more than existing native plants in wetlands. A recent study in the Yancheng salt marsh of China found that *S*. *alterniflora* invasion led to three times more SOC and more than twice as much root biomass, but no significant difference in soil respiration between *S*. *alterniflora* and *Phragmites australis* stands [16]. This may be due to continuous surface water inundation restricting soil respiration across stands; however, additional research is needed to explain the lack of soil respiration differences. More generally, the effects of plant invasion on in situ soil respiration in wetland ecosystems are not well understood.

*S*. *alterniflora* invasion into China’s coastal wetlands provides an excellent opportunity to study plant invasion impacts on soil respiration. Native to United States Atlantic and Gulf Coasts, *S*. *alterniflora* is an intertidal brackish and saltmarsh plant species. It has become a global invasive species, rapidly spreading in coastal wetlands in the Pacific coast of North America, Europe, New Zealand, and China [17]. In 1979, *S*. *alterniflora* was intentionally introduced to China’s east coast to promote sediment accumulation and land formation [18]. Since then, *S*. *alterniflora* has rapidly expanded and displaced native plant species, forming large monocultures. *S*. *alterniflora* is now found in most Chinese coastal areas from Tianjin (38°56'N, 121°35'E) in the north, to Beihai (21°36'N, 109°42'E) in the south [19].

Prior to *S*. *alterniflora* invasion into the Yangtze River estuary, *P*. *australis* and *Scirpus mariqueter* were the two dominant native C_3_ plant species in the high and low tide zones, respectively [20]. As a C_4_ species, *S*. *alterniflora* has a number of superior traits over the native plant species, including faster growth, greater productivity, and denser root system [18]. *S*. *alterniflora* produces overwintering ramets in autumn/winter, while the two native ones emerge only in spring [21]. All these factors give *S*. *alterniflora* competitive advantages over the two native plant species. Additionally, *S*. *alterniflora* tolerates high salinity and water inundation better than the two native plant species [22]. *S*. *alterniflora* has rapidly displaced *P*. *australis* in the high tide zone and *S*. *mariqueter* in the low tide zone in Yangtze River estuary, with several consequences [18]. *S*. *alterniflora* invasion has significantly increased plant biomass and soil total carbon (TC) [21], altered microbial diversity [23], changed nematode trophic groups [24] and litter decomposition [25]. These factors are known to impact soil respiration; however, these impacts have not been fully researched.

This study examined how *S*. *alterniflora* invasion affects soil respiration and C dynamics, and whether invasion-induced changes in soil respiration are significantly different between the high tide zone and the low tide zone of the Yangtze River estuary coastal wetlands. The study involved measuring in situ soil respiration rates along two transects that were originally occupied by two native plant species, but have been invaded by *S*. *alterniflora*. We also examined differences in aboveground NPP (ANPP), SOC, SMBC, and soil properties between *S*. *alterniflora* stands and the two high and low tide native plant stands.

## Materials and Methods

### Ethics Statement

The study was carried out in a coastal wetland on Chongming Island, Shanghai, managed by the Dongtan National Natural Reserve Administration. No specific permission was required to access this land, and field studies did not involve any endangered or protected species.

### Study Site

**Fig 1:**
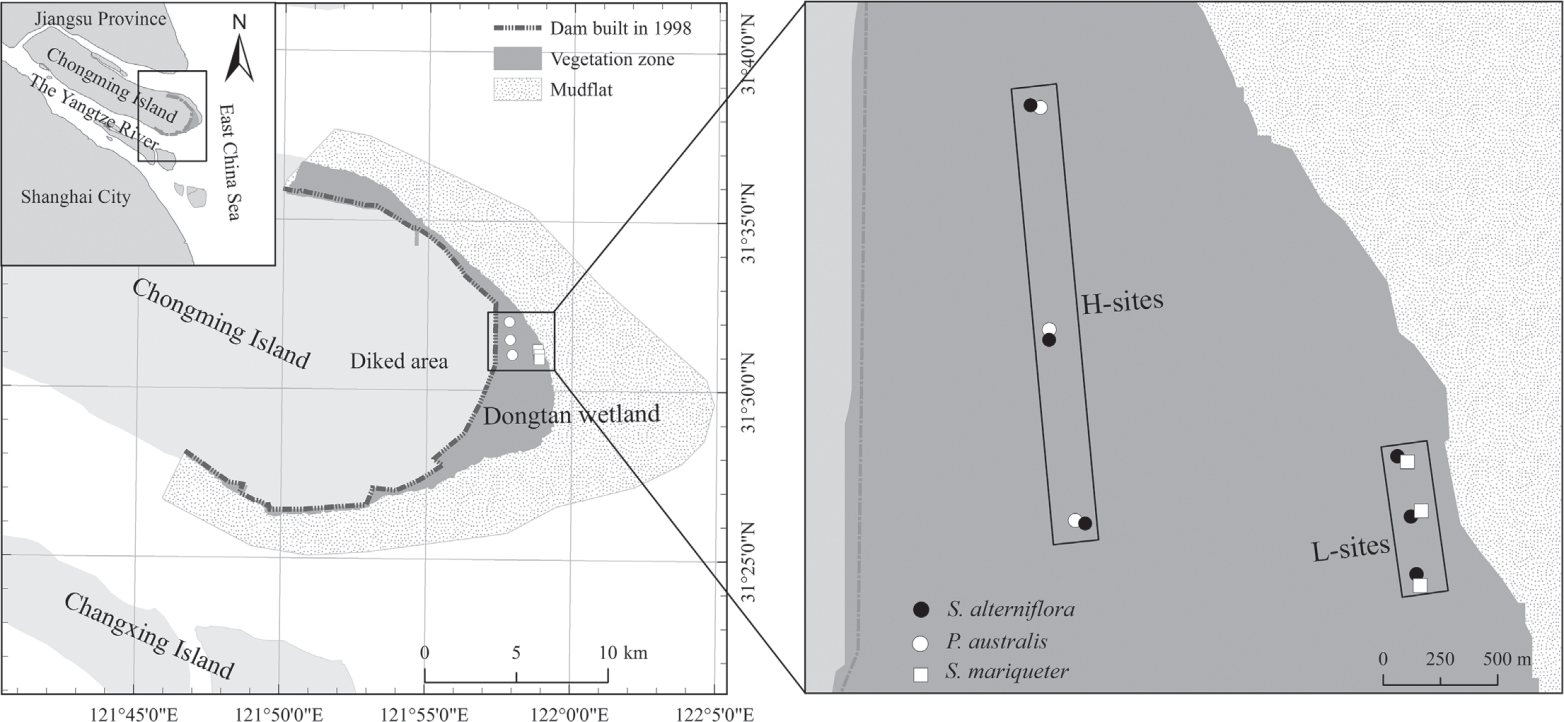
Locations of sampling transects and sites in the Dongtan wetland of Chongming Island, the Yangtze River estuary, China. The study area was drawn using ArcGIS software, with remote sensing data set MOD09Q1of 2009 downloaded from NASA Earth Observatory without copyright restrictions.

The study sites were at the Dongtan wetland on Chongming Island in the Yangtze River estuary, China (31°25'‒31°38'N, 121°50'‒122°05' E) (Fig. 1). The wetland covers approximately 230 km^2^, and is expanding annually at a rate of about 150–200 m in distance, or 4.06 km^2^ in area [26]. The wetland has a typical subtropical monsoon climate, with mean annual precipitation of 1022 mm, and mean temperature of 15.3°C [20].

The Dongtan wetland elevation decreases gradually from a dike built in 1998 to the sea; it can be divided into high, middle, and low tidal zones [27]. The low tide zone has a higher salinity, larger soil particle size, and more frequent and longer tidal inundation periods than the high tide area [20]. *P*. *australis* and *S*. *mariqueter* were the native dominant plant species in the Dongtan wetland before *S*. *alterniflora* invaded. *P*. *australis* is a C_3_ perennial grass of Poaceae, forms monocultures, and thrives in the high tide zone, at a relatively high elevation. *S*. *mariqueter*, an endemic species of China, is a C_3_ perennial bulrush. It is mainly found in the low tide zone at a relatively low elevation. *S*. *alterniflora*, a perennial rhizomatous C_4_ grass of Poaceae, was introduced to the northern Dongtan wetland in 2001. The ideal salinity for *S*. *alterniflora* to grow and reproduce ranges from 8 to 33‰ [17]. Currently, *S*. *alterniflora* is widely distributed in wetland vegetation, and its monocultures accounted for 49.4% of Dongtan wetland’s vegetated area in 2005 [18].

### Sampling transects and experimental design

We established two sampling transects parallel to the dike, one in the high tide zone and one in the low tide zone (Fig. 1). Three sites were evenly distributed on each transect. The high tide zone transect, including three sites (H-sites), was 2.5 km long; the low tide zone transect, including three sites (L-sites), was 0.8 km long. Closely adjacent stands were selected at each site: *Spartina*-*Phragmites* was located at the H-sites, and *Spartina*-*Scirpus* was located at the L-sites. There were approximately 8-meters distance between *S*. *alterniflora* and the native plant stands at each site. In each of the adjacent stands, three plots were randomly selected for gas, plant, and soil sampling. All stands were consistent monocultures, and were larger than 10 m in diameter. This experimental design minimized the potential effects of heterogeneous environmental conditions, such as tidal inundation in the wetland.

### Gas sampling and analysis

Soil respiration was measured using a static close chamber method [6]. The plexiglass chamber (thickness: 7mm) had a dimension of 40 × 40 × 45 cm. A thermometer installed inside the chamber measured air temperature. A stainless steel tube with a 2 mm internal diameter balanced air pressure inside and outside the chamber. A battery-driven fan was installed on one inside wall of the chamber to mix air. To sample gas from inside the chamber, a 22 cm long needle was fixed in the middle of the chamber’s top wall. The sampling needle collected gas at the chamber’s center; the upper end was connected to a three-way valve. A aluminum foil was wrapped around the chamber to minimize internal air temperature changes during measurements [28].

Before each field sampling event, the aboveground plants and litter in the measuring chamber area were regularly clipped and removed, without disturbing the surface soil [29]. The sampling chambers were gently placed on and pressed into the soil surface, creating a seal between the chamber and the soil while minimizing disturbance. While a base collar is often used with this sampling tool, in this case, a collar would have changed the sedimentation and soil water condition, and was not used. A perfect seal between the chamber and the soil was easily achieved because of the soft surface soil. The four corners of each 45 × 45 cm sampling plot was marked by bamboo poles to maintain the same chamber position for each sampling event.

Gas sampling were conducted in November 2008 and January, February, March, April, May, August, and October 2009 for H-sites, and in May, August, October, November 2009 and January 2010 for L-sites. Gas sampling was not conducted in September 2009 because of excessive rain during neap tides. For each sampling event, gas samples were collected during neap tide period, when vegetation zones were not inundated by tidewater for several days and the soil surface was exposed to the air. Gas sampling was conducted between 10:00 and 15:00 on sunny days.

Approximately 50 ml of gas was collected using syringes 10, 20, 30, and 40 minutes after the chamber was closed. The first 10 minutes allowed the chamber to achieve a stable state [30]. All gas samples were quickly injected into pre-evacuated vials after collection and analyzed in the laboratory as soon as possible [31].

In addition to gas sampling, soil temperature was measured at a depth of 5 cm near the chamber, and a soil sample (0–5 cm) was collected close to each chamber to determine soil moisture content. Gas sample CO_2_ concentrations were determined using a gas chromatography system (Agilent 6890N, Agilent Company, USA) equipped with a FID detector (200°C). The CO_2_ was separated by a Porapak-Q packed column (2 m length, running at 25 ml/min, and 55°C) and transformed to CH_4_ by a nickel catalyst reformer (375°C). For the analysis phase, the gas sample CO_2_ concentrations were plotted against time; CO_2_ efflux was determined using an incremental concentration slope.

### Plant and soil sampling and analysis

Plant biomass was sampled in all plots in September 2010, when the aboveground plant biomass was the highest, based on a previous study in a nearby wetland [21]. All aboveground plant biomass was harvested from a 50 × 50 cm area at each stand and then oven-dried at 50°C to a constant weight to determine C content.

All soil samples were collected using stainless steel tubes with an inner diameter of 5 cm and a length of 110 cm. To determine total C (TC) and SOC, soil cores were collected from each site in May 2009 and divided into seven sections based on depth (0–5, 5–10, 10–20, 20–40, 40–60, 60–80, and 80–100 cm). Soil was sampled at a depth of 0–20 cm in May, August, and November 2009 and January 2010 to measure soil microbial biomass C (SMBC), and sampled again in June 2010 to determine soil pH.

Soil samples were oven-dried at 105°C to a constant weight to determine gravimetric moisture [32]. Soil pH was measured in the laboratory using a multi-parameter water quality analyzer (YSI 556 MPS, YSI Incorporated, USA) and a 1:2.5 slurry (weight/volume, soil: distilled water) [32]. Plant C and soil TC were analyzed using an elemental analyzer (FlashEA 1112 Series, Thermo Fisher Scientific, USA). Dried soil samples were treated with 1 N HCl for 24 h at room temperature to remove inorganic carbon (SIC) [33]. SOC was determined using a TOC analyzer (Multi N/C 3100 with solid module HT1300, Analytik Jena AG, Germany). SIC was estimated by subtracting SOC from TC [34]. SMBC was measured using a chloroform (CHCl_3_) fumigation-extraction method with 0.5 M K_2_SO_4_ after Wu et al. [35]. The organic carbon content of the K_2_SO_4_ extract was analyzed using a TOC analyzer (Multi N/C 3100, Analytik Jena AG, Germany). SMBC was calculated from the increase in extractable C in the fumigated soils compared with the controls, using a universal conversion factor of 0.45 [35].

### Data analyses

First, we analyzed the differences in soil respiration, temperature, and moisture between paired stands on the same transect (i.e. *S*. *alterniflora* vs. *P*. *australis* stands in the H-sites, *S*. *alterniflora* vs. *S*. *mariqueter* stands in the L-sites) using repeated-measures ANOVA. *T*-tests were used to analyze differences in ANPP, soil pH, TC, SOC, SIC, and mean SMBC between paired stands on the same transect. Second, *t*-tests were also used to determine differences in ANPP, time-weighted mean of soil respiration, mean moisture, SOC, SIC, and mean SMBC between *S*. *alterniflora* stands in the H- and L- sites. Third, differences in ANPP, time-weighted mean of soil respiration, SOC, and mean SMBC between *S*. *alterniflora* and native plant species in the H- and L- sites were examined using *t*-tests. All data were tested against assumptions of normality and equal variances. Post-hoc Duncan tests examined differences between means (*P* < 0.05). All statistics were calculated using SPSS 13.0 for Windows.

## Results

### Soil respiration

**Fig 2:**
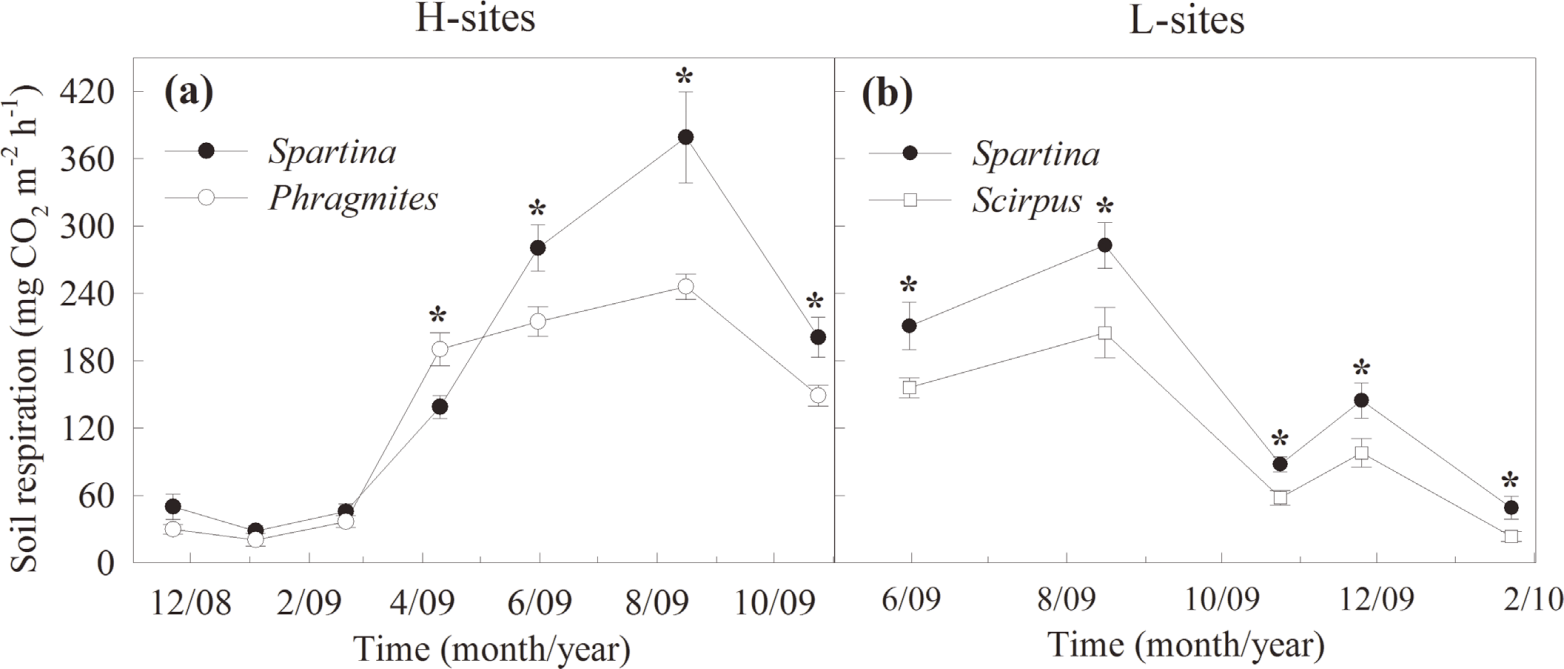
Temporal variations in soil respiration in different plant stands. Temporal variations in soil respiration under *S*. *alterniflora* (*Spartina*) and *P*. *australis* (*Phragmites*) stands in the H-sites, and *S*. *alterniflora* and *S*. *mariqueter* (*Scirpus*) stands in the L-sites. Bars represent mean ± SE (n = 9), asterisks indicate significant differences (*P* < 0.05) between means of different stands.

Soil respiration rates were significantly higher in *S*. *alterniflora* than *P*. *australis* stands in the H-sites (Table 1), with mean values of 185.8 ± 7.1 and 142.3 ± 9.8 mg CO_2_ m^−2^ h^−1^, respectively (Fig. 2A). In the L-sites, the mean soil respiration rate in the *S*. *alterniflora* stands was 159.7 ± 7.4 mg CO_2_ m^−2^ h^−1^ during the study period, which was significantly higher than in the *S*. *mariqueter* stands (112.0 ± 8.2 mg CO_2_ m^−2^ h^−1^, Table 1, Fig. 2B). In all stands, soil respiration was lowest in January and highest in August (Table 1, Fig. 2). The time-weighted mean of soil respiration rate in the *S*. *alterniflora* stands in the H-sites was significantly higher than in the L-sites (*T*_1, 16_ = 6.5, *P* = 0.021). Similarly, soil respiration in the *P*. *australi* in the H-sites was also significantly higher than in the *S*. *mariqueter* in the L-sites (*T*_1, 16_ = 6.0, *P* = 0.026). The difference in mean soil respiration between *S*. *alterniflora* and *P*. *australis* stands in the H-sites (43.5 ± 11.5 mg CO_2_ m^−2^ h^−1^) was similar to between *S*. *alterniflora* and *S*. *mariqueter* stands in the L-sites (47.8 ± 12.3 mg CO_2_ m^−2^ h^−1^; *T*_1, 16_ = 0.07, *P* = 0.803).

**Table 1:** Results of repeated-measures ANOVA for effects of stands, sampling time, and their interaction on soil respiration, temperature, and moisture between paired stands (i.e. *S*. *alterniflora* and *P*. *australis* stands in the H-sites, *S*. *alterniflora* and *S*. *mariqueter* stands in the L-sites) for different times.

			Soil respiration		Temperature		Moisture	
Sites	Source of variation	*df*	*F value*	*P value*	*F value*	*P value*	*F value*	*P value*
H-sites	Stand	1, 16	12.5	0.003	0.6	0.804	9.0	0.013
	Sampling time	6, 11	108.7	3.800E-9	1654.2	6.148E-19	2.8	0.054
	Stand ×Sampling time	6, 11	6.2	0.005	0.3	0.304	0.2	0.400
L-sites	Stand	1, 16	17.6	0.001	0.003	0.956	7.8	0.013
	Sampling time	4, 13	85.7	3.302E-9	1437.0	7.126E-62	23.3	6.227E-19
	Stand ×Sampling time	4, 13	1.2	0.375	1.41	0.240	1.1	0.269

*Notes*: H-sites and L-sites refer to high-tide-zone sites and low-tide-zone sites, respectively.

### Aboveground net primary production

In both the H- and L-sites, the ANPP was significantly higher in the *S*. *alterniflora* than in the native plant stands (Tables 1, 2). In the H-sites, the ANPP of *S*. *alterniflora* stands was significantly higher than in the L-sites (*T*_1, 16_ = 9.0, *P* = 0.008; Table 2). The difference in ANPP between *S*. *alterniflora* and *S*. *mariqueter* stands in the L-sites was significantly larger than between *S*. *alterniflora* and *P*. *australis* stands in the H-sites (*T*_1, 16_ = 6.0, *P* = 0.026).

**Table 2:** Aboveground net primary production (ANPP), soil pH, total carbon (TC), organic and inorganic carbon (SOC and SIC, 0–100 cm) and SMBC in different stands and tide sites.

	H-sites		L-sites	
Variables	*S*. *alterniflora*	*P*. *australis*	*S*. *alterniflora*	*S*. *mariqueter*
ANPP (kg C m^−2^)	0.82 (0.02) ^a^	0.29 (0.01) ^b^	0.94 (0.05) ^a^	0.18 (0.01) ^b^
Soil pH	8.80 (0.04) ^a^	8.76 (0.05) ^a^	8.66 (0.03) ^a^	8.65 (0.02) ^a^
TC (mg g^−1^)	15.9 (0.2) ^a^	15.0 (0.3) ^b^	15.0 (0.1) ^a^	13.4 (0.2) ^b^
SOC (mg g^−1^)	5.2 (0.2) ^a^	4.5 (0.2) ^b^	4.3 (0.1) ^a^	2.8 (0.2) ^b^
SIC (mg g^−1^)	10.6 (0.2) ^a^	10.5 (0.3) ^a^	10.7 (0.1) ^a^	10.6 (0.1) ^a^
SMBC (mg kg^−1^)	76.0 (7.0) ^a^	45.0 (5.3) ^b^	45.4 (5.4) ^a^	30.0 (5.1) ^b^

*Notes*: H-sites and L-sites refer to high-tide-zone sites and low-tide-zone sites, respectively. Numerical values are means (±SE), different letters indicate significant differences (*P* < 0.05) between means of different stands.

### Soil properties and carbon content

**Fig 3:**
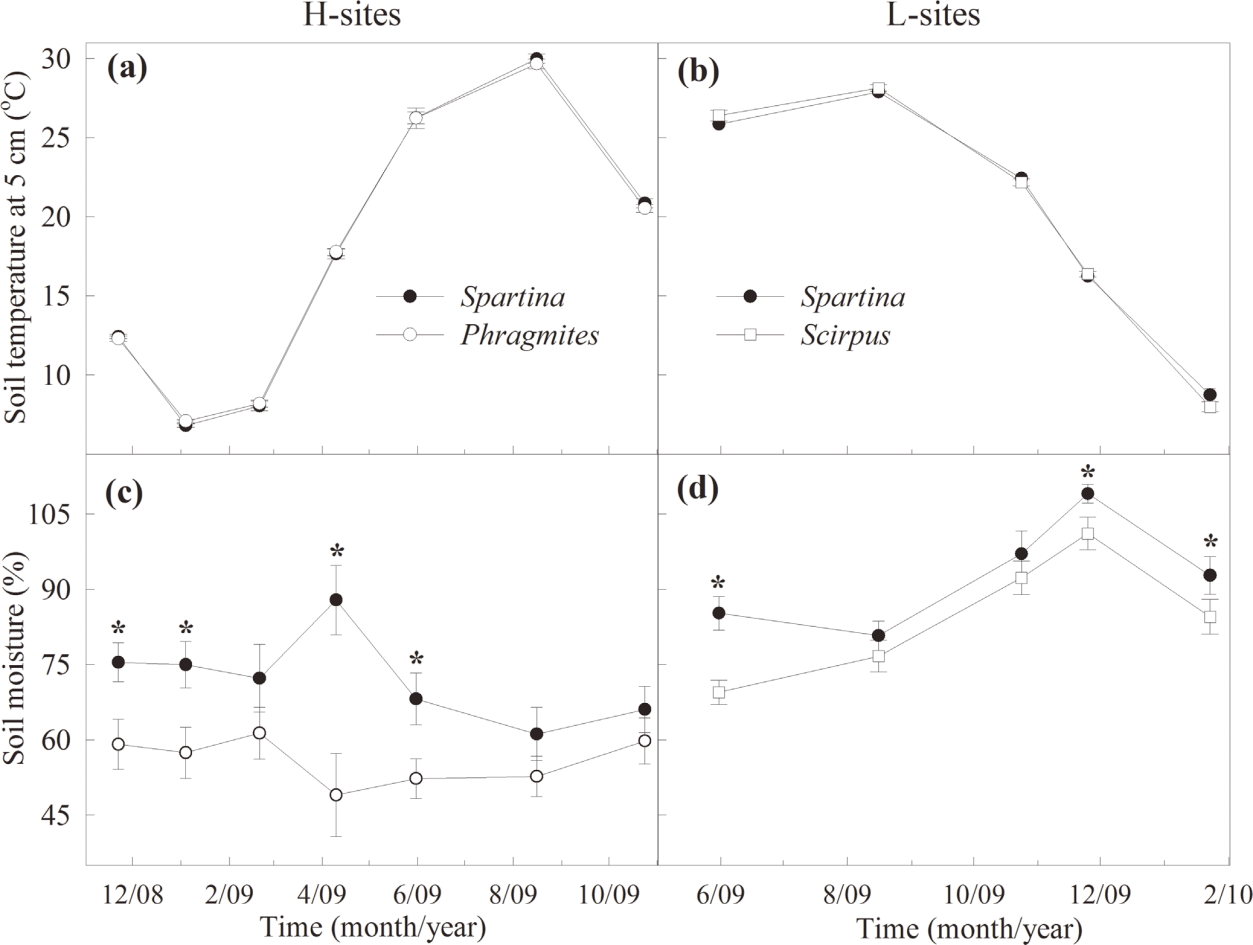
Temporal variations in soil temperature and moisture in different plant stands. Temporal variations in soil temperature (a, b) and moisture (c, d) at 0–5 cm depth in the *S*. *alterniflora* (*Spartina*) and *P*. *australis* (*Phragmites*) stands in the H-sites, *S*. *alterniflora* and *S*. *mariqueter* (*Scirpus*) stands in the L-sites. Bars represent mean ± SE (n = 9), asterisks indicate significant differences (*P* < 0.05) between means of different stands.

Soil temperature in the *S*. *alterniflora* stands did not significantly differ from the native plant stands, with the lowest value in January and the highest in August (Table 1, Fig. 3A, 3B). Soil moisture was significantly higher in the *S*. *alterniflora* stands than in the native stands in both the H- and L-sites (Table 1, Fig. 3C, 3D). In the H-sites, mean soil moisture was significantly lower than in the L-sites (*T*_1, 34_ = 72.4, *P* = 2.370E-7). Soil pH was not affected by plant invasion or site locations (Tables 2, 3).

**Table 3:** Results of *t*-test for effects of different stands on aboveground net primary production (ANPP), soil pH, total carbon (TC), organic and inorganic carbon (SOC and SIC, 0–100 cm), and SMBC.

		H-sites		L-sites	
Variables	*df*	*T value*	*P value*	*T value*	*P value*
ANPP	16	111.3	1.253E-13	32.6	2.821E-11
Soil pH	16	0.4	0.563	0.03	0.876
TC	16	6.8	0.019	50.3	2.538E-6
SOC	16	6.0	0.026	56.6	1.224E-6
SIC	16	0.6	0.446	1.9	0.188
SMBC	16	23.2	0.009	16.0	0.016

*Notes*: H-sites and L-sites refer to high-tide-zone sites and low-tide-zone sites, respectively.

**Fig 4:**
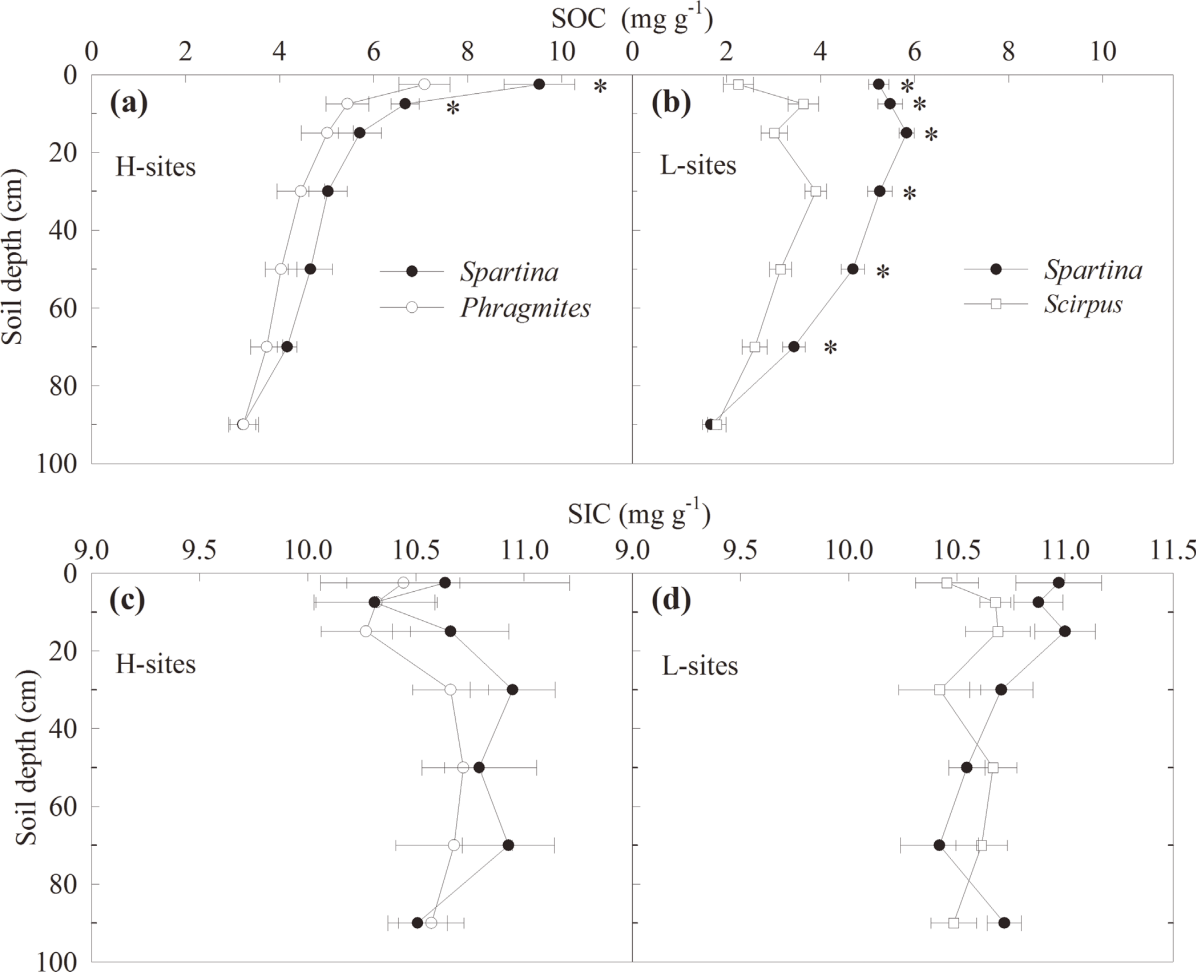
Soil organic and inorganic carbon in different plant stands. Soil organic carbon (SOC) and inorganic carbon (SIC) distribution in soil profiles (0–100 cm deep) in the *S*. *alterniflora* (*Spartina*) and *P*. *australis* (*Phragmites*) stands in the H-sites, and *S*. *alterniflora* and *S*. *mariqueter* (*Scirpus*) stands in the L-sites. Bars represent mean ± SE (n = 9), asterisks indicate significant differences (*P* < 0.05) between means of different stands.

Soil TC and SOC (0–100 cm) were both significantly higher in the *S*. *alterniflora* than in the two native plant stands (Tables 2, 3). However, statistically significant differences in SOC were only detected between *S*. *alterniflora* and *P*. *australis* stands in the H-sites topsoil (0–10 cm) (*T*_1, 16_ = 6.4, *P* = 0.022; Fig. 4A); there were also statistically significant differences in SOC between *S*. *alterniflora* and *S*. *mariqueter* stands in L-sites’ 0–80 cm soil layer (*T*_1, 16_ = 79.8, *P* = 1.290E-7; Fig. 4B). In the H-sites, SOC in the *S*. *alterniflora* stands was significantly higher than that in the L-sites (*T*_16_ = 16.6, *P* = 0.001; Table 2). In the Dongtan wetland, the proportion of SIC to soil TC was 60.4% and 63.1% in the *S*. *alterniflora* and *P*. *australis* stands in the H-sites, and 72.9% and 79.2% in the *S*. *alterniflora* and *S*. *mariqueter* stands in the L-sites, respectively (Table 2). There were no significant difference in SIC between plant species or site locations (Tables 2, 3, Fig. 4C, 4D).

**Fig 5:**
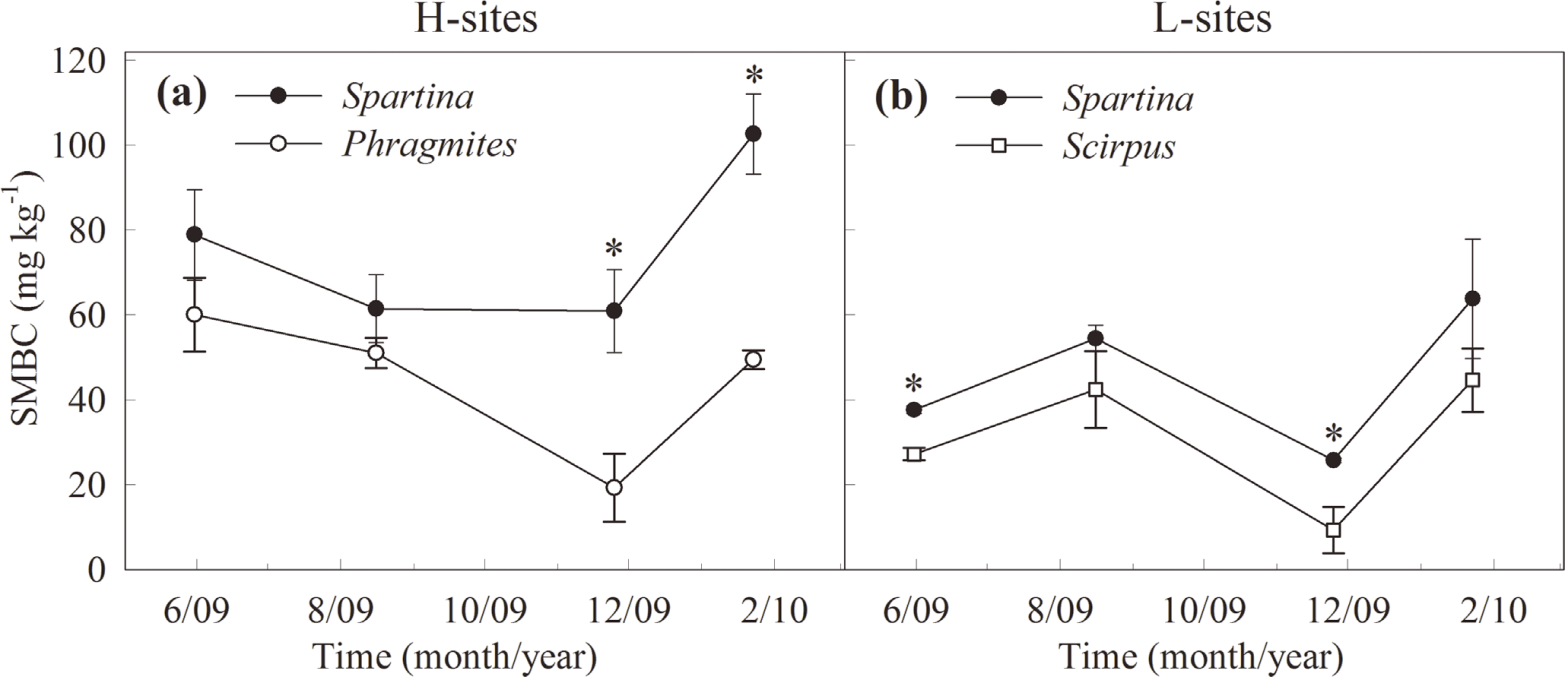
Soil microbial biomass carbon in different plant stands. Seasonal variations in soil microbial biomass carbon (SMBC) content (0–20 cm deep) in the *S*. *alterniflora* (*Spartina*) and *P*. *australis* (*Phragmites*) stands in the H-sites, and *S*. *alterniflora* and *S*. *mariqueter* (*Scirpus*) stands in the L-sites. Bars represent mean ± SE (n = 9), asterisks indicate significant differences (*P* < 0.05) between means of different stands.

In both the H- and L-sites, the mean SMBC in the *S*. *alterniflora* stands was significantly higher than in the native plant stands (Tables 2, 3, Fig. 5). Furthermore, the SMBC in the *S*. *alterniflora* stands was significantly higher in the H-sites than in the L-sites (*T*_1, 16_ = 21.3, *P* = 0.010; *P* < 0.05, Fig. 5).

## Discussion

### Effects of *S*. *alterniflora* invasion on soil respiration

There are only three dominant species in the Dongtan wetland: *P*. *australis*, *S*. *mariqueter*, and *S*. *alterniflora* [18]. Each forms a monoculture, because of relatively homogeneous ambient sediment. However, microenvironment heterogeneities may create uncertainties about the estimated effects of *S*. *alterniflora* invasion on soil respiration. In our study, pair-wised sampling and repeated measures to compare the *S*. *alterniflora* and the two native plant stands reduced these uncertainties. A pairwise design between invasive and native plots is a common practice to investigate invasion effects [7, 8, 21, 36, 37].

Soil respiration is mainly regulated by SOC quantity and quality, root and microbe biomass and activities, soil temperature and moisture, and by soil pH in some circumstances [6, 10]. This study found that *S*. *alterniflora* invasion significantly enhancined soil respiration in the Yangtze River estuary; several mechanisms may drive increases in both microbial and root respiration. First, *S*. *alterniflora* has a longer growing season and a higher net photosynthetic rate than *P*. *australis* and *S*. *mariqueter* [21, 36], generating a higher ANPP and more organic C in the soil. *S*. *alterniflora* also significantly increased soil labile C pools [38], supplying more substrates (e.g. root exudates, litter, and available SOC) to the soil microbes, leading to increased microbial biomass (SMBC, Fig. 5), and enhanced microbial respiration. Second, the root biomass in the *S*. *alterniflora* stands was significantly higher than in the native plant stands [21], leading to higher root respiration. Finally, although increased soil moisture can restrict O_2_ diffusion from the atmosphere into the soil [39], this negative effect on soil respiration may not be significant. This is because soil moisture is high and O_2_ diffusion is uniformly restricted in coastal wetlands. All three species in this study are vascular plants with the ability to transport O_2_ into the soil through roots [21, 40]. However, the significantly higher root biomass of *S*. *alterniflora* transports more O_2_ into the soil compared with *P*. *australis* and *S*. *mariqueter*. Thus, *S*. *alterniflora* invasion may increase soil respiration by improving soil O_2_ supply. Overall, the higher soil respiration in the *S*. *alterniflora* stands across tide zones can be attributed to an enhanced ANPP in *S*. *alterniflora* compared to the native plant species.

Compared to *P*. *australis* and *S*. *mariqueter*, *S*. *alterniflora* has lower quality litter, due to its lower nitrogen content [25, 41]. This may decrease the C release rate from litter decomposition and weaken soil respiration. Previous studies, however, have reported that *S*. *alterniflora* invasion can improve soil nitrogen status through two mechanisms. First, the *S*. *alterniflora* stem and sheath litter nitrogen content increases significantly along with decomposition process; this is a result of N_2_ fixation of epiphytic microbial communities, leading to higher soil nitrogen. This phenomena is not seen in the litter of the two native plant species [25, 42]. Second, *S*. *alterniflora* intercepts and takes up tidal nitrogen subsidies more than the two native plant species [37]. This gradually improved nitrogen status during the litter decomposition. This, combined with the higher quantity of *S*. *alterniflora* litter, can induce more C release from litter [25], stimulating soil respiration. In addition, Zhang et al. [43] showed that the decomposition of mixed litter from invasive and native plants can increase soil respiration at intermediate invasion stages. However, *S*. *alterniflora* is rapidly excluding and displacing *P*. *australis* and *S*. *mariqueter* in the Dongtan and other wetlands of Yangtze River estuary [17]. The three dominant plant species currently form their own monocultures, resulting in no invasive and native litter mixing. Given this, there are likely no significant effects from litter mixture on soil respiration in the case of *S*. *alterniflora* invasion in our study area.

Soil respiration in the *S*. *alterniflora* stands was significantly higher in the H-sites than in the L-sites. This difference may be due to higher SOC and SMBC and lower soil moisture in the high tide zone (Figs. 3–5). Further, our results showed that *S*. *alterniflora* soil respiration increases were more uniform in the H- sites than in the L-sites. This may be because the higher soil moisture in the low tide zone may limit soil microbial activities, although increased SOC by *S*. *alterniflora* invasion was higher in the H-sites than in the L-sites.

On the United States east coast, *P*. *australis* has invaded and replaced native species *S*. *alterniflora* and *S*. *patens* [44, 45]. There, *P*. *australis* stands have been reported to have a significantly higher NPP than *S*. *patens* [46] and *S*. *alterniflora* stands [47]. However, SOC in the *P*. *australis* stands was similar or lower than *S*. *alterniflora* stands [47, 48]. No study has reported the effects of *P*. *australis* invasion on in situ soil respiration on the United States east coast. Based on our study of *S*. *alterniflora* invasion in the Yangtze River estuary, we speculate that *P*. *australis* invasion into United States coastal wetlands may also enhance soil respiration, because *P*. *australis* has significantly increased C input to the soil, but has not significantly change soil C pools. Further comparative studies of *S*. *alterniflora* invasion into the Yangtze River estuary and *P*. *australis* into U.S. coastal wetlands could yield greater insights about the effects of plant invasion on belowground C processes in the context of global change.

### *S*. *alterniflora* invasion and soil C content

*S*. *alterniflora* invasion has significantly increased ANPP in both the H- and L-sites (Table 2); however, the increase in ANPP in the L-sites was significantly higher than in the H-sites. Two possible mechanisms may explain this. First, inorganic nitrogen availability is a major limiting factors for plant growth in salt marshes [49]. In the Dongtan wetland, *S*. *alterniflora* can intercept and uptake more inorganic nitrogen from tidewater than the two native species [37]. This advantage is more prominent in the L-sites because of more frequent and longer inundation periods at these sites [50]. Second, *S*. *alterniflora* growth can be restricted when previous-year litter blocks incoming light [51]. In the Dongtan wetland, tidewater washed away more litter in the L-sites than in the H-sites, supporting better *S*. *alterniflora* growth in the low tide zones. In addition, a lower ANPP baseline before low tide zone invasion was also an important contributor.

SOC accumulation in the *S*. *alterniflora* stands has been promoted primarily by increased above- and below-ground biomass/NPP [21, 52]. In coastal wetlands, plants play an important role in trapping sediments [53, 54]. Previous studies conducted in the Dongtan wetland have found that sedimentation rate in the *S*. *alterniflora* stands was significantly higher than in *P*. *australis* and *S*. *mariqueter* stands [55, 56]. In the H-sites, SOC was highest in the surface layer and declined gradually with soil depth (Fig. 4A). This may be due to a relatively slow sedimentation in the high tide zone [55]. In the L-sites, the highest SOC was found at a 5–10 cm depth in the *S*. *mariqueter* stand and at a 10–20 cm depth in the *S*. *alterniflora* stand. This suggests that *S*. *alterniflora* sedimentation in the low tide zone helps bury organic C faster into the soil. The interaction between *S*. *alterniflora* invasion and sediment deposition is critical to SOC accumulation in Yangtze River estuary wetlands.

The estimated SIC in the Dongtan wetland, which accounts for more than 60% of TC in the soil profile to a depth of 100 cm, was consistent with a previous study conducted in a nearby Jiuduansha wetland [57]. SIC can be affected by vegetation type and soil properties, such as soil pH, [34, 58]. *S*. *alterniflora* invasion may decrease soil pH probably through exudation of low-molecule-weight organic acids from roots [59] and preferential absorption of NH_4_^+^-N [60]. However, periodic tidal inundation can maintain an alkaline environment (pH > 8.0) in Yangtze River estuary soil [61]. Soil TC serves as an indicator of the effects of *S*. *alterniflora* invasion on soil C dynamics [21, 56]. The lack of difference in SIC between *S*. *alterniflora* and the native plant stands suggested that TC in coastal wetland soils does not reflect invasion effects.

### Implications

It is predicted that increases in sea level will accelerate in the future if current global climate change continues [62]. Rises in sea level may significantly impact on coastal wetland ecosystems, changing species composition and reducing wetland ecosystems stability [63]. The vertical accretion of coastal wetlands through accumulating mineral and organic materials help mitigate these impacts [64]. When compared with native plant species *P*. *australis* and *S*. *mariqueter*, the invasive *S*. *alterniflora* can trap more tidewater minerals and produce more organic matter [56], contributing to tidal flat accretion. The accretion may be higher in the low tide zone because of higher plant organic matter input, lower organic matter decomposition (indicating by lower soil respiration), and greater frequency and duration of tidal inundation periods, transporting mineral materials in the low tide zone. In this manner, *S*. *alterniflora* invasion may help stabilize coastal wetland ecosystems in the Yangtze River estuary as the climate change, with greater impacts in the low tide zone than in the high tide zone. Additional study is needed to assess whether enhanced sediment accretion by *S*. *alterniflora* can keep pace with accelerating sea level rises.

Coastal wetlands are important net C sinks [65]. The effects of *S*. *alterniflora* invasion into Yangtze River estuary wetlands on ecosystem C cycling was dominated by significantly increased ANPP. In wetland ecosystems, increased ANPP enhances C sequestration and strengthen C sink, because organic C decomposition is kept low by the high soil moisture, especially in the low tide zone.

*S*. *alterniflora* is a C_4_ plant species; the two native plant species *P*. *australis* and *S*. *mariqueter* are C_3_ plants [17]. Our findings suggest that the success of *S*. *alterniflora* invasion into the Yangtze River estuary was mainly due to a higher ANPP compared to its native counterparts. C_4_ plant species perform better than C_3_ species in stressful environments, such as in hot and/or drought conditions [66, 67]. This enables C_4_ species to achieve a higher NPP, which gives advantages in resource competition and tolerances. In the Yangtze River estuary, however, the success of *S*. *alterniflora* invasion may be unrelated to C_4_ and C_3_ species differences. As a parallel case, *P*. *australis* has successfully invaded *S*. *alterniflora* on the United States east coast. Both *S*. *alterniflora* in the Yangtze River estuary and *P*. *australis* in the United States have higher NPP than the native species. The mechanisms underpinning the enhanced productivities of *S*. *alterniflora* in the Yangtze River estuary and *P*. *australis* in United States coastal wetlands are critical to improve plant invasion control.

## Conclusions

*S*. *alterniflora* invasion into Yangtze River estuary coastal wetlands has significantly increased ecosystem ANPP and SOC, and has enhanced soil microbial activities by increasing microbial biomass and substrate supply. Soil respiration was higher in the *S*. *alterniflora* stands than in the native plants stands; the net effects of *S*. *alterniflora* invasion is to strengthen coastal wetland’s C sink. Compared to the native plant species *P*. *australis* and *S*. *mariqueter*, *S*. *alterniflora* speeds the burial of plant-derived organic matter into subsoil by increasing sediment deposition. This may supplement SOC accumulation in invaded Yangtze River estuary wetland ecosystems.
